# Rural-urban variation in hypertension among women in Ghana: insights from a national survey

**DOI:** 10.1186/s12889-021-12204-7

**Published:** 2021-11-24

**Authors:** Francis Appiah, Edward Kwabena Ameyaw, Joseph Kojo Oduro, Linus Baatiema, Francis Sambah, Abdul-Aziz Seidu, Bright Opoku Ahinkorah, Eugene Budu

**Affiliations:** 1 Department of Population and Health, Faculty of Social Sciences, University of Cape Coast, Cape Coast, Ghana; 2 Department of Social Sciences, Berekum College of Education, Berekum, Bono Region Ghana; 3 School of Public Health, Faculty of Health, University of Technology Sydney, Sydney, NSW 2007 Australia; 4 Ghana Health Service, Upper West Regional Health Directorate, Wa, Ghana; 5 Department of Health, Physical Education and Recreation, University of Cape Coast, Cape Coast, Ghana; 6 College of Public Health, Medical and Veterinary Sciences, James Cook University, Townsville, Queensland Australia; 7 Department of Estate Management, Takoradi Technical University, Takoradi, Ghana; 8 Centre For Gender and Advocacy, Takoradi Technical University, P. O. Box 256, Takoradi, Ghana

**Keywords:** Ghana, Hypertension, Rural, Urban, Women, DHS

## Abstract

**Background:**

Hypertension is one of the leading causes of cardiovascular morbidities in Ghana and represents a major public health concern. There is dearth of information on the rural-urban disparity in hypertension among women in Ghana. Therefore, this study aimed at examining the rural-urban variation in hypertension among women in Ghana.

**Methods:**

We extracted data from the women’s file of the 2014 Ghana Demographic and Health Survey. The sample included 9333 women aged 15–49 with complete data on hypertension. The analysis was done using Pearson Chi-square and binary logistic regression at 95% confidence interval. The results of the binary logistic regression were presented as Odds Ratios (ORs) and Adjusted Odds Ratios (AORs). Statistical significance was set at *p* < 0.05.

**Results:**

Hypertension prevalence among urban and rural residents were 9.5% and 5.1% respectively. Rural women had lower odds of hypertension [OR = 0.59; 95% CI = 0.52, 0.67] compared to urban women, however, this was insignificant in the adjusted model [aOR = 0.84; 95% CI = 0.70, 1.00]. The propensity to be hypertensive was lower for women aged 15–19 [aOR = 0.07; 95% CI = 0.05, 0.11]. The poorest were less likely to be hypertensive [aOR = 0.63; 95% CI = 0.45, 0.89]. Single women were also less probable to have hypertension [aOR = 0.66; 95% CI = 0.46, 0.97].

**Conclusions:**

Women from urban and rural areas shed similar chance to be hypertensive in Ghana. Therefore, the health sector needs to target women from both areas of residence (rural/urban) when designing their programmes that are intended to modify women’s lifestyle in order to reduce their risks of hypertension. Other categories of women that need to be prioritised to avert hypertension are those who are heading towards the end of their reproductive age, richest women and the divorced.

## Background

Hypertension is one of the leading causes of cardiovascular morbidity, mortality and chronic kidney diseases and represents a serious public health challenge [1]. It accounts for 33% of global preventable premature deaths and disability [2]. Globally, hypertension is estimated to cause 7.5 million deaths, signifying about 12.8% of all deaths annually [3]. In most high-income countries, it is recorded as the primary cause of death and was a major contributory factor in over 250,000 out of the 2.4 million deaths in 2017 [4]. Hypertension is also one of the leading risk factors of health challenges in low-and-middle-income countries [5]. About 1.13 billion people worldwide are hypertensive and most (two-thirds) are in low- and middle-income countries **[**6**].** In 2015, 1 in 5 women were hypertensive globally **[**6**].**

Although the proportion of the world’s population with uncontrolled hypertension fell modestly between 1980 and 2008, the number of people with this condition rose from 600 million in 1980 to nearly 1 billion in 2008 due to population growth and ageing [3]. It is projected that 17.4 million people will have hypertension, due to increase in population between 2015 and 2030 [7], if the necessary functional and effective preventive measures are not put in place to cater for this major health challenge in the low-and-middle-income countries [2]. In particular, the prevalence of hypertension is on the increase in Ghana [2]. In rural and urban areas of Ghana, the prevalence of hypertension ranges between 19% and 48% respectively with some studies reporting 24% or higher in rural areas [2].

Several studies have concluded that the high increase in hypertension is associated with changes in dietary patterns, sedentary lifestyles and preventive risky health behaviours, which have been shown to differ based on whether an individual lives in a rural or urban residence [8–10]. Notwithstanding the rural-urban disparity in hypertension globally, few studies in Ghana have explored the factors that account for the disparity. While efforts to explain hypertension-related issues have focused on socio-demographic characteristics [11–15], little attention have been paid to the rural-urban discrepancy in hypertension at the national level [16, 17]. Other studies suggest that history and prevalence of hypertension are associated with socio-demographic characteristics in both rural and urban areas [2, 11, 12, 18].

The dearth of information on the rural-urban disparity in hypertension in Ghana [2, 19] presents significant impediment in targeted areas, functional and effective treatment and prevention of hypertension in the rural-urban areas of Ghana. Hence, this study comprehensively examined the rural-urban variation in hypertension among women in Ghana. Our study targeted only women because existing studies have either investigated only men or both sexes and hypertension disparities among the rural-urban areas [20–24]. These studies are silent on the rural-urban variation in hypertension among women only. Therefore, this study examined the rural-urban difference in hypertension using data from the 2014 Ghana Demographic and Health Survey (GDHS) with focus on women. Understanding the disparities in hypertension among the rural-urban populace of women in Ghana is important for developing national strategies to better prevent and control hypertension through collaborative national efforts. Improving the management and control of hypertension in the face of limited resources necessitate strategic strategies for preventative interventions that target behavioural change through education, as well as functional and effective policy execution.

## Methods

### Source of data

In this study, we used data from the 2014 GDHS. Since the inception of the GDHS, it is only the 2014 edition that assessed hypertension status of Ghanaian women. The GDHS is a five-year interval nationally representative survey mostly carried out by the Demographic and Health Surveys (DHS) Program, Ghana Statistical Service and Ghana Health Service [25]. The survey seeks to collect, analyse, and circulate representative and reliable data on core health indicators in over 90 countries including Ghana. These core healthcare indicators comprise adult health and lifestyle including hypertensive status, nutrition as well as maternal and child health. In 2014, the survey recruited 9396 women within the 15–49 age group. The survey made use of an updated frame prepared for the 2010 Population and Housing Census (PHC) and had a response rate of 97%. We included 9333 women in the present study because this sample had complete information for the analysis. The sample was derived through a two-stage sampling approach aimed at permitting estimation of core indicators throughout the then 10 administrative regions. The first stage involved the selection of sample points or clusters made up of enumeration areas (EAs) whereas the second stage constituted a sampling of households following systematic sampling. Between January and March 2014, household listing was conducted for this purpose. Approximately 30 households were identified per cluster. This resulted in 427 clusters (with 216 from urban and 211 from rural settings) and 12,831 households throughout the country [25]*.* The sample excluded institutional and nomadic persons such as those in hotels and prisons. The data was deemed suitable for the study because it is nationally representative and the first of its kind to investigate the hypertensive status of women in their reproductive age at the national level. We had access to the dataset from the website of Measure DHS and is freely available through https://dhsprogram.com/data/available-datasets.cfm.

### Dependent variable

Hypertensive status (measured by blood pressure) of Ghanaian women aged 15–49 was the dependent variable for the study. During the 2014 DHS, blood pressure was monitored and measured on three occasions following the UA-767F/FAC (A&D Medical) blood pressure with at least 10 min interval [25]. In determining hypertensive status, an average of the second and third measurements were computed, and this conforms to calibration by other studies on hypertension that are underpinned by the DHS datasets [16, 26, 27]. Following the Joint National Committee Seven (JNC7) guideline, hypertension was conceptualised as an average systolic blood pressure of ≥140 mmHg and/or an average diastolic blood pressure of ≥90 mmHg. Hypertensive women were coded as 1 whilst non-hypertensive women were coded otherwise ‘0’.

### Explanatory variables

The main explanatory variable was place of residence (rural or urban), in line with the categorisation of the DHS survey. The choice of this explanatory variable emerged from its statistically significant association with hypertension with dominance among the urban population [18, 28, 29] whilst some evidence also documents high inclination toward rural residents [9]. There was, therefore, the need to interrogate and identify the situation in Ghana. We included some socio-demographic characteristics of the women; age, wealth quintile, marital status, occupation, salted fish consumption, region and tobacco use (comprising cigarette, tobacco, and snuff). We included salted fish consumption because some evidence indicates an association between hypertension and salt intake [30, 31]. Behavioural factors such tobacco use and some related lifestyles have been documented as precursors to hypertension [32–34]. The following variables were recoded to suit the analysis; marital status was recoded as “single = 0”, “married = 1”, “cohabiting = 2”, “widowed = 3”, “divorced = 4” and “separated=5; occupation recoded into “not working=0” “agriculture = 1″ “manual = 2″ and “service = 1″; salted fish consumption into “No = 0″ and “Yes = 1.”

### Data analyses

**Fig. 1:**
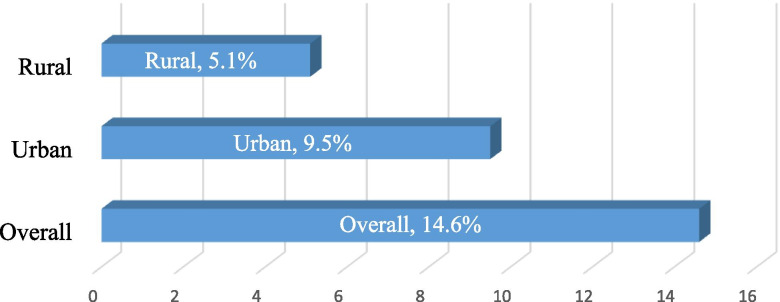
Rural/urban distribution of hypertension among women

In our analysis, we calculated the proportion of women with hypertension by place of residence (rural or urban) as shown in Fig. 1. We also computed the proportion of hypertension by the socio-demographic variables as shown in Table 1, and also explored which of them had a significant association with hypertension. Out of the ten variables tested (see Table 1), six were significant and were used in our inferential analysis (residence, age, wealth, marital status, occupation, and region). To ensure that there is no multicollinearity between the explanatory variables, tests for multicollinearity was conducted and it was revealed that the socio-demographic variables are not highly correlated [mean VIF = 1.42, maximum = 2.31, minimum = 1.02]. Due to the dichotomous nature of our dependent variable, binary logistic regression analysis was conducted where odds ratios (ORs) and adjusted odds ratios (aORs) with their respective 95% confidence intervals (95% CI) were reported (Table 2). Model 1 focused on the bivariate analysis between residence and hypertension whilst Model 2 presents a multivariable model adjusting for the effect of the significant socio-demographic variables. The results were weighted in order to achieve proportionality at the national level and the entire analysis was conducted using Stata version 13.

**Table 1:** Socio-demographic characteristics and hypertension among Ghanaian women (*n* = 9333)

Variable	Sample n %		Hypertension Urban Rural n(%) n(%)	
***Age (X***^***2***^ ***= 463.590; p < 0.001)***				
15–19	1621	17.3	55.5	44.5
20–24	1599	17.2	66.4	33.6
25–29	1597	17.1	65.0	35.0
30–34	1362	14.6	71.2	28.8
35–39	1283	13.8	66.6	33.4
40–44	1022	11.0	65.8	34.2
45–49	849	9.1	61.0	39.0
***Wealth quintile (X***^***2***^ ***= 22.185; p < 0.001)***				
Poorest	1505	16.1	13.8	86.2
Poorer	1630	17.5	19.2	80.8
Middle	1924	20.6	50.8	49.2
Richer	2099	22.5	81.2	18.8
Richest	2175	23.3	97.9	2.1
***Marital Status (X***^***2***^ ***= 241.297; p < 0.001)***				
Single	3081	33.0	72.9	27.1
Married	3936	42.2	64.2	35.8
Cohabiting	1342	14.4	58.5	41.5
Widowed	250	2.7	66.0	34.0
Divorced	280	3.0	61.8	38.2
Separated	444	4.7	69.5	30.5
***Education (X***^***2***^ ***= 850.914; p < 0.001)***				
No education	1782	19.1	40.9	59.1
Primary	1664	17.8	58.2	41.8
Secondary	5300	56.8	72.8	27.2
Higher	587	6.3	85.6	14.4
***Occupation (X***^***2***^ ***= 70.488; p < 0.001)***				
Not working	2183	23.4	64.3	35.7
Agriculture	1746	18.7	18.7	81.3
Manual	1146	12.3	68.4	31.6
Service	4258	45.6	77.5	22.5
***Salted fish consumption (X***^***2***^ ***= 0.350; p = 0.554)***				
No	6011	64.4	66.3	33.7
Yes	3322	35.6	63.1	36.9
***Region (X***^***2***^ ***= 37.553; p < 0.001)***				
Western	1037	11.1	41.5	58.5
Central	932	10.0	47.0	53.0
Greater Accra	1888	20.2	92.2	7.8
Volta	717	7.7	36.8	63.2
Eastern	867	9.3	54.3	45.7
Ashanti	1775	19.0	71.9	28.1
Brong Ahafo	766	8.2	63.9	36.1
Northern	783	8.4	36.6	63.4
Upper East	357	3.8	34.9	65.1
Upper West	211	2.3	44.2	55.8
***Tobacco use (X***^***2***^ ***= 0.266, p = 0.606)***				
No	9295	99.6	65.2	34.8
Yes	38	0.4	28.6	71.4

Source: 2014 GDHS

**Table 2:** Binary logistic regression results on residential status and hypertension in Ghana

Variable	Model I		Model II	
	OR	95% CI	aOR	95% CI
***Place of residence***				
Urban	1	[1,1]	1	[1,1]
Rural	0.59^***^	[0.52,0.67]	0.84	[0.70,1.00]
***Age***				
15–19			0.074^***^	[0.05,0.11]
20–24			0.10^***^	[0.07,0.13]
25–29			0.17^***^	[0.13,0.22]
30–34			0.28^***^	[0.23,0.36]
35–39			0.46^***^	[0.37,0.56]
40–44			0.61^***^	[0.50,0.75]
45–49			1	[1,1]
***Wealth quintile***				
Poorest			0.63^**^	[0.45,0.89]
Poorer			0.73^*^	[0.55,0.98]
Middle			0.83	[0.66,1.05]
Richer			0.80^*^	[0.65,0.98]
Richest			1	[1,1]
***Marital Status***				
Single			0.66^*^	[0.46,0.97]
Married			0.71^*^	[0.52,0.98]
Cohabiting			0.71	[0.50,1.00]
Widowed			0.81	[0.54,1.23]
Divorced			1	[1,1]
Separated			0.80	[0.53,1.19]
***Education***				
No education			0.94	[0.68,1.31]
Primary			1.12	[0.82,1.54]
Secondary			1.05	[0.79,1.39]
Higher			1	[1,1]
***Occupation***				
Not working			1.14	[0.89,1.48]
Agriculture			0.84	[0.66,1.08]
Manual			1	[1,1]
Service			1.13	[0.93,1.39]
***Region of residence***				
Western			1.70^**^	[1.18,2.47]
Central			1.51^*^	[1.04,2.20]
Greater Accra			2.17^***^	[1.50,3.13]
Volta			1.95^***^	[1.35,2.82]
Eastern			1.50^*^	[1.03,2.17]
Ashanti			1.92^***^	[1.34,2.76]
Brong Ahafo			1.68^**^	[1.16,2.41]
Northern			1.29	[0.89,1.87]
Upper East			1.13	[0.77,1.66]
Upper West			1	[1,1]
pseudo *R*^2^	0.010		0.123	
***N***	**9333**		**9333**	

Exponentiated coefficients; 95% confidence intervals in brackets, OR = Odd Ratio, aOR = Adjusted Odds Ratio, ^*^ *p* < 0.05, ^**^ *p* < 0.01, ^***^ *p* < 0.001, 1 = Reference categorys

Source: 2014 GDHS

### Ethical approval

DHS reports that informed consent was sought from all the women prior to their participation in the survey. The DHS sought ethical approval from the Ethics Committee of ORC Macro Inc. and that of Ghana Health Service. Authors of this manuscript were not directly involved in the data collection processes but rather obtained access by applying to the DHS (via https://dhsprogram.com/data/available-datasets.cfm) in order to obtain access.

## Results

### Hypertension prevalence

The findings indicate that 14.6% of the participants had hypertension nationwide. Of these, 9.5% emerged from urban locations whilst the remaining 5.1% occurred among women in rural residential areas of Ghana.

### Socio-demographic characteristics and hypertension among Ghanaian women

As indicated in Table 1, 71.2% of urban women aged 30–34 had hypertension with 55.5% of those aged 15–19 women also having it. Nearly all the richest urban residents had hypertension (97.9%), whilst only 13.8% poorest women in urban Ghana had hypertension. Single women in urban Ghana had hypertension (72.9%) and most women who had secondary education and resided in urban settings had hypertension (72.8%). With respect to occupation, 77.5% of those in service profession and living in urban Ghana had hypertension, however, only 18.7% of those in urban settings in agriculture had hypertension. A significant proportion of women in urban locations who were not consuming salted fish were hypertensive (66.3%). Across the regions, at least nine out of ten urban residents in the Greater Accra region had hypertension (92.2%). Hypertension is quite phenomenal among urban women who use tobacco (71.4%). With the exception of tobacco use and salted fish consumption, the rest of the socio demographic characteristics were significantly associated with hypertension at 95% significance level.

### Binary logistic regression results on residential status and hypertension in Ghana

Outcome of the binary logistic regression is presented in Table 2. In the crude model (model I), rural women had lower odds of hypertension [OR = 0.59; 95% CI = 0.52, 0.67] compared to urban women, however, this was insignificant in the adjusted/final model. The propensity to be hypertensive was lower for women aged 15–19 [aOR = 0.074; 95% CI = 0.051, 0.11] relative to those aged 45–49. Compared to the richest women, poorest women were less likely to be hypertensive [aOR = 0.63; 95% CI = 0.45, 0.89]. Single women were less probable to experience hypertension as compared with the divorced [aOR = 0.66; 95% CI = 0.46, 0.97]. We also realised that, compared to women in the Upper West region, women of all other regions were more probable to be hypertensive particularly those in the Greater Accra region [aOR = 2.17; 95% CI = 1.50, 3.13].

## Discussion

This study sought to find out the difference between urban and rural female populations with regards to hypertension in Ghana. The major finding was that residential status of women (i.e. rural/urban) was not a determinant of hypertension in the present study. However, theoretically significant covariates such as age, wealth quintile, marital status and region of residence influenced the likelihood to be hypertensive. These suggest that other socio-demographic characteristics such as age and behavioural factors are important contributors to hypertension [35–37]. It is worth noting that at the bivariate level, rural residents had lower odds to hypertension and this was significant. This indicates that originally, rural women had lower chances of hypertension. However, this was ﻿attenuated when we controlled for other factors (i.e. the covariates). Perhaps, rural residence are gradually taking up lifestyles similar to those in urban locations in terms of diet and exercise [38].

The analysis also revealed that women between 45 and 49 years had higher odds of having hypertension. This result is in consonance with findings by Kafle and colleagues [37] who indicated that the likelihood to suffer from hypertension increases as one advances in age. Similarly, Peltzer and Phaswana-Mafuya [35], noted that older participants had higher odds of hypertension compared with younger ones and this persisted after controlling for confounding variables. Additionally, a multi-country study among developing and developed countries showed that positive association with increasing age and body mass index corresponds to a higher chance of being hypertensive [36]. Buford [39] synthesised some diverse complex mechanisms such as inflammation, oxidative stress, endothelial dysfunction and indicated that advancement in age plays some mechanistic functions in the development of cardiovascular conditions and increases the risk of hypertension later on in life. This could explain why the aged were inclined to hypertension.

We realised that poorer women had a lower likelihood to be hypertensive as compared to the richest. This is similar to the observation made by an earlier study in Ghana [40]. Plausibly, the richest might have been exposed to sedentary lifestyle which inclines the richest to be hypertensive as opposed to the poorer [41, 42]. However, the results contradict findings by Lloyd-Sherlock et al. [36] who reported that hypertension was more common among those in the lowest wealth quintile. This could be due to differences in other socio-demographic characteristics of women who were surveyed in the present study and their responses.

In furtherance, the study revealed that single women were less inclined to develop hypertension as compared to e divorced women. A plausible explanation for this could be explained on the grounds that the person is experiencing possible emotional instability. The psychosocial distress associated with losing one’s partners might have compelled the divorced and widowed to resort to some hypertension inclined lifestyles as a coping mechanism. Finally, we also found that residing in the Greater Accra, Central, Volta and Western regions (i.e. regions closer to the Atlantic Ocean) increased the chance to suffer from hypertension as compared to staying in the Upper West region. Scholars have remarked that proximity to seashore is associated with high salt intake (sodium chloride) arising from the consumption of drinking water containing salt exceeding the recommended limits [43]. At the same time, coastal dwellers’ agricultural products including cereals, fruits, vegetables and sea food may have excess salt content which also predispose them to high salt consumption [43].

Considering the proximity of these regions to the sea and availability of sea food, this could suggest that there is a higher consumption of salted fish and other sea food which are fortified with sodium. Although sodium is a major nutrient obtained from salt, the World Health Organisation recommends a level of sodium intake less than 2 g per day for adults in order to reduce blood pressure, risk of cardiovascular diseases, stroke and coronary heart disease [44, 45]. If someone goes beyond the recommended threshold, it will render such a person susceptible to adverse outcomes. Residents in the Ashanti, Eastern and Brong Ahafo regions (non-coastal regions), showed findings similar to coastal regions. We admit that, the cross-sectional nature of our dataset limits the effort to reveal the reasons behind this observation. Perhaps, women in the Eastern, Ashanti and Brong Ahafo regions might have been exposed to risky behaviours such as less intake of fruits, alcohol consumption, and lack of physical exercise [34, 46–48].

### Strengths and limitations

The conclusions drawn for the study are based on the larger sample size derived by probabilistic method used, hence, having a true representation of the population studied. A weakness of the study is that due to the cross-sectional nature of the survey, causality could not be established. Also, the study only reflected women’s hypertension situation, hence the conclusions drawn may not be applicable for men. Finally, the study methodology, which is cross-sectional in nature, limited the effort to explore reasons behind some of our observations.

## Conclusions

Our study has indicated that rural/urban differential does not really matter as far as women’s propensity to hypertension is concerned in Ghana. Therefore, the Ghana Health Service through the Health Promotion and Education unit needs to target women from both residences (rural/urban) with their programmes designed to reduce risks of hypertension. Other categories of women that need to be prioritised to avert hypertension are those who are heading towards the end of their reproductive age, richest women and the divorced.

## Data Availability

The datasets generated and/or analysed in this study are available in the Measure DHS repository, www.measuredhs.org.

## Abbreviations

aOR: Adjusted Odds Ratio
DHS: Demographic and Health Surveys
EAs: Enumeration Areas
GDHS: Ghana Demographic and Health Survey
JNC7: Joint National Committee Seven
OR: Odds Ratios
PHC: Population and Housing Census
